# The Personalization of Conversational Agents in Health Care: Systematic Review

**DOI:** 10.2196/15360

**Published:** 2019-11-07

**Authors:** Ahmet Baki Kocaballi, Shlomo Berkovsky, Juan C Quiroz, Liliana Laranjo, Huong Ly Tong, Dana Rezazadegan, Agustina Briatore, Enrico Coiera

**Affiliations:** 1 Australian Institute of Health Innovation Faculty of Medicine and Health Sciences Macquarie University Sydney Australia; 2 Health Information Systems Office, Ministry of Health Buenos Aires Argentina

**Keywords:** conversational interfaces, conversational agents, dialogue systems, personalization, customization, adaptive systems, health care

## Abstract

**Background:**

The personalization of conversational agents with natural language user interfaces is seeing increasing use in health care applications, shaping the content, structure, or purpose of the dialogue between humans and conversational agents.

**Objective:**

The goal of this systematic review was to understand the ways in which personalization has been used with conversational agents in health care and characterize the methods of its implementation.

**Methods:**

We searched on PubMed, Embase, CINAHL, PsycInfo, and ACM Digital Library using a predefined search strategy. The studies were included if they: (1) were primary research studies that focused on consumers, caregivers, or health care professionals; (2) involved a conversational agent with an unconstrained natural language interface; (3) tested the system with human subjects; and (4) implemented personalization features.

**Results:**

The search found 1958 publications. After abstract and full-text screening, 13 studies were included in the review. Common examples of personalized content included feedback, daily health reports, alerts, warnings, and recommendations. The personalization features were implemented without a theoretical framework of customization and with limited evaluation of its impact. While conversational agents with personalization features were reported to improve user satisfaction, user engagement and dialogue quality, the role of personalization in improving health outcomes was not assessed directly.

**Conclusions:**

Most of the studies in our review implemented the personalization features without theoretical or evidence-based support for them and did not leverage the recent developments in other domains of personalization. Future research could incorporate personalization as a distinct design factor with a more careful consideration of its impact on health outcomes and its implications on patient safety, privacy, and decision-making.

## Introduction

### Background

Recent advancements in natural language recognition and synthesis have resulted in the adoption of conversational agents (CAs) in many fields. CAs can be defined as systems that support conversational interaction with users by means of speech or other modalities [1]. The rising popularity of conversational technologies has been facilitated by a renaissance in Artificial Intelligence, the development of powerful processors supporting deep learning algorithms, and technological advancements, making a large amount of computationally accessible knowledge available [1].

One emerging area in which conversational technologies have been increasingly used is health care. A recent systematic review in this area examined technical performance, user experience, and health-related outcomes and found that most studies had not employed standardized evaluation methods or had failed to address aspects of patient safety [2]. There have also been other recent reviews on health care conversational agents [3-5]. This study differs from them in that it focuses on the implementation of personalization in health care conversational agents.

### Personalization

Personalization is:

the process of making something suitable for the needs of a particular person [6].

When applied specifically to digital technologies, personalization can be defined as:

a process that changes the functionality, interface, information access and content, or distinctiveness of a system to increase its personal relevance to an individual or a category of individuals [7].

A recent interdisciplinary review study proposed a framework to characterize personalization along three dimensions: (1) what is personalized (ie, content, user interface, delivery channel, and functionality); (2) for whom is it personalized (either a specific individual or a user group, eg, elderly women); and (3) how automated is the personalization (how the information needed for user modelling is collected) [7]. The personalization process involves user models containing characteristics, preferences, interests, and needs of users as the basis for providing adaptive information and services. Depending on the degree of automation, two types of personalization can be distinguished: implicit and explicit. In implicit personalization, information needed for user models is obtained automatically through the analysis of observed user activities and interactions with the system. In explicit personalization, information needed for user models requires users’ active participation in obtaining the required information.

### Personalization in Conversational Agents

One of the earliest applications of personalization in a conversational system was Grundy, a virtual librarian that delivered book recommendations [8]. To build a user model for personalization purposes, Grundy asked questions at the beginning of an interaction and associated users with predefined stereotypes. After the initial user provided information, the user model was updated implicitly over time during conversations. It was a hybrid system bringing together both explicit and implicit personalization. This foundational work on personalized CAs has been followed by a range of works focusing on dialogue management [9], personalized messages [10], recommender systems [11], and adaptive systems [12].

Personalization in CAs can be achieved implicitly by processing past interactions with users [11,13] or explicitly by user-entered information at the set-up time [8] or using ongoing confirmation style input [14]. The messages presented to users [10], or the conversational style of systems [15], can be personalized. Personalized and adaptive system behavior in conversational systems can improve user comprehension [16], user satisfaction [17], task efficiency [18], and the likelihood of behavior change [19]. Furthermore, personalization can be an essential system feature for voice interfaces due to the limitations in presenting large amounts of information through a voice-only modality [20]. The effects of personalization have been evaluated in various ways by measuring aspects like efficiency in terms of the number and duration of interactions [11,20], user satisfaction, relevance, and understandability [20], information quality presented [21], and appropriateness of system responses [10].

### Personalization in Health Care and Medicine

Studies of personalization in health care and medicine have been increasing in number since the early 2000s [22], with growing evidence showing their effectiveness [23-26]. One important limitation in the health care personalization literature is equating it to genomics-supported efforts in medicine [27]. Genomic markers are only one dimension of personalization that helps to recognize the uniqueness of individuals and make their medicine personalized [27,28]. There are other factors that affect this personalization of health care, such as people’s lifestyle choices, their socioeconomic context and living environment, and other health care services that can be personalized like health education and therapies [29].

A review of behavior change interventions characterized four intervention groups according to their degree of personalization in the messages delivered to individuals: generic (one-size-fits-all messages), personalized (messages with the person’s name), targeted (messages specific to a subgroup of the general population), or tailored (messages specific to an individual’s characteristics) [30]. The review found that 78% (11/14) of the tailored and 95% (22/23) of the targeted interventions reported improved outcomes, with 54% (6/11) of the tailored and 68% (15/22) of the targeted interventions being statistically significant.

Dialogue systems can offer fine-grained possibilities to personalize the information to be delivered:

on the basis of the inferred goals and beliefs of the user at a particular moment in time, and incorporating everything that has previously been said in the conversation [31].

Learning from a history of previous conversations plays a key role in ensuring the continuity of health communications that take place over multiple interactions over time [31].

Informed by the recent theoretical developments in personalization [7], a broader understanding of personalization in health care [22,29], and an increasing interest in health care CAs [3-5], this study aims to review the use of personalization in health care CAs and characterize the methods that have been applied to implement this personalization. Aligned with the rapid advancements in natural language processing technologies used in CAs [1] and the increasing adoption of CAs using unconstrained natural language [32], this review focuses on agents with unconstrained natural language input capability. These agents include chatbots, which can engage in small talk or casual dialogues, embodied conversational agents, which feature computer-generated visual virtual characters capable of both verbal and nonverbal communication, and commonly available smart conversational interfaces such as Apple’s Siri, Google’s Google Assistant, Samsung’s Bixby, and Microsoft’s Cortana [1,33,34].

## Methods

### Overview

This review uses the search protocol of an earlier systematic review that was performed between April 2017 and February 2018, with a focus on technical performance, user experience, and the health-related outcomes of CAs in health care [2]. The current review has: (1) focused on the use of personalization features in CAs that were not examined previously; (2) used the same inclusion and exclusion criteria as the review by Laranjo et al [2] with an additional criterion on personalization (ie, the studies with no personalization features were excluded); and (3) performed a new search in March 2019.

### Search Strategy

We searched in the PubMed, Embase, CINAHL, PsycInfo, and the ACM Digital Library databases, and did not restrict by the publication year or language. The search terms included “conversational agents”, “dialogue systems”, “relational agents” and “chatbots”. The complete search strategy is available in Multimedia Appendix 1. In addition, the reference lists of relevant articles and grey literature identified in those databases were also included for screening.

### Study Selection Criteria

The identified publications were included if they: (1) were primary research studies that focused on consumers, caregivers, or health care professionals; (2) involved a conversational agent; and (3) tested the system with human users. The studies were excluded if they involved: (1) user input by clicking or tapping an answer amongst a set of predefined choices, or by using the telephone keypad (eg, interactive voice response systems with dual tone multi frequency); (2) output not generated in response to what it received from the human user (eg, predefined and preprogrammed messages that are not dependent on the information obtained from or about the user); (3) question-answer type interactions; (4) asynchronous communication technology such as email; or (5) no personalization features. Furthermore, studies evaluating only individual components of a conversational agent, like automatic speech recognition, or using Wizard of Oz methods were excluded.

### Screening, Data Extraction, and Synthesis

Screening was conducted independently by two researchers to extract data from each study. Cohen kappa was used to measure intercoder agreement between the researchers. Any disagreements between the assessments of two researchers were resolved by consensus agreement. To identify the relevant information, the researchers used the personalization definition presented in the introduction section. In addition, the following keywords were used as a guide to identify personalization-related information within the studies: personalizing, adapting, customizing, tailoring, configuring, individualizing, modifying, changing, altering, transforming, modelling, tuning, setting, preference, and profile. The data extraction process was guided by an assessment scheme based on the personalization framework offered by Fan and Poole [7]. In addition to these dimensions, we included three more dimensions to provide further details on the included studies: purpose of personalization, methods to evaluate personalization, and outcomes in relation to personalization. Table 1 summarizes the final assessment scheme for personalization.

**Table 1:** An assessment scheme for personalization.

Assessment categories			Description
**Automation^a^**			How the user models needed by personalization are constructed.
	Implicit	Information needed for user models is obtained automatically through the analysis of observed user activities and interactions with the system (eg, analyzing users’ conversational history to determine the suitable times to send a reminder).	
	Explicit	Information needed for user models requires users’ active participation in obtaining the required information (eg, selecting the preferred times to receive a reminder).	
**Target^a^**			For whom to personalize.
	Individuated	Personalization is targeted at a specific individual (eg, sending a reminder based on the unique profile of a single user).	
	Categorical	Personalization is targeted at a group of people (eg, sending a reminder based on a shared profile of a group of users).	
**Aspects of the system^a^**			What to personalize.
	Content	The information itself (eg, alerts or reminders).	
	User interface	How the information is presented (eg, using larger font sizes for elderly users or shortening prompts for experienced users).	
	Delivery channel	The media through which information is delivered (eg, sending a reminder as a text message instead of a voice message).	
	Functionality	What users can do with the system (eg, making different system functionalities available for patients and carers).	
Purpose			The purpose of personalization (eg, increasing user engagement or motivation).
Evaluation			The methods to evaluate personalization (eg, using interview questions or standardized questionnaires).
Outcomes			The outcomes in relation to personalization (eg, increased user engagement or motivation).

^a^Adapted from Fan and Poole [7].

## Results

### Search Results

**Figure 1:**
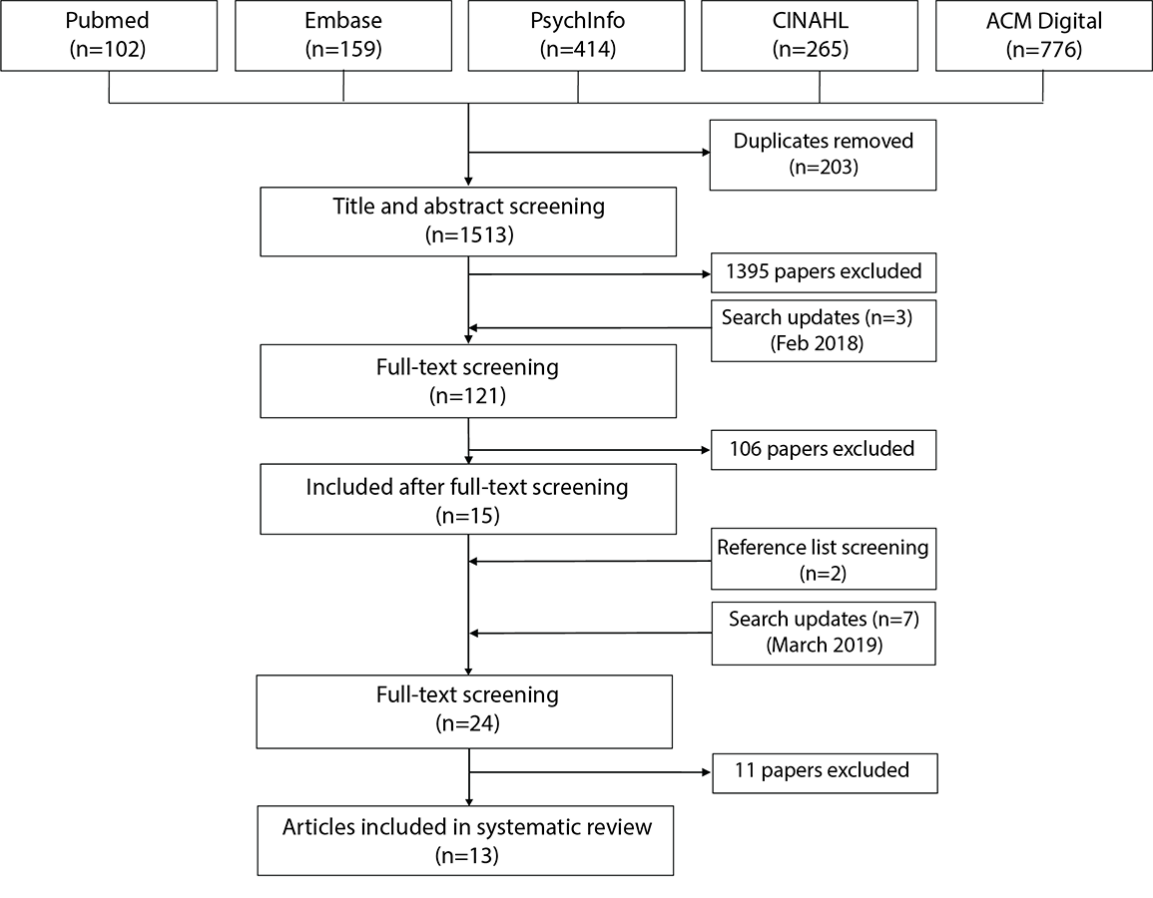
Preferred Reporting Items for Systematic Reviews and Meta-Analyses flow diagram.

The first search found 1513 papers, and the updated search found an additional 445 papers (Figure 1). After the subsequent title, abstract and full text screenings, 13 studies were included in this review [35-47]. The first search’s kappa statistic for the title and abstract screening was 0.45 (fair agreement) and for the full-text screening it was 0.53 (fair agreement). For the updated search, the kappa score was 0.77 for the title and abstract screening (substantial agreement), and 0.61 for the full-text screening (substantial agreement). The list of excluded studies, their major themes, and the reasons for their exclusion are available in Multimedia Appendix 2.

### Implementation of Personalization Features and Target Population

Table 2 and Table 3 summarize the personalization features of CAs in the included studies [35-47]. For both tables, studies evaluating the same conversational agent were grouped together. Since the delivery channel and functionality were not personalized by any of the studies, they were not included in Table 2. Out of the 13, 8 studies supported patients and 5 studies supported both patients and clinicians. Regarding the target of personalization, all the studies implemented individuated personalization (targeting an individual user). However, one study employed categorical personalization (targeting a group of people) to differentiate novice and expert users in addition to the individuated one [41].

### Automation of Personalization

Information needed for personalization was provided explicitly by the users in seven studies [35,37-40,44,45], and obtained implicitly by the system in one study [36] where the conversational agent analyzed users’ audio-visual features, such as facial expression and head position, to determine its feedback. A mix of implicit and explicit methods was employed by five studies [41-43,46,47]. Across all the studies, data explicitly entered by the users included personal goals [35,37,46,47], symptoms and medications [37,44,45], measurement of vital signs [39,40], knowledge level on a specific topic [38], and daily practices [38]. User data implicitly obtained by the systems primarily involved the analysis of conversation history. Differently from the rest, one study analyzed users’ voice and nonverbal facial gestures to determine narrative skills of the users [36].

**Table 2:** Personalization features of conversational agents in the included studies.

Conversational agent (author, year)	CA^a^ purpose	Automation (the basis for personalization)	Target (for whom to personalize)	What to personalize	
				Content	User interface
Tess (Fulmer et al, 2018) [44]	Delivery of cognitive behavioral therapy to reduce symptoms of depression and anxiety in college students	Explicit: Expressed emotions and mental health concerns of participants to provide personalized responses. Users' feedback and reported mood used to tailor interventions	Individuated	Personalized conversations based on emotions and mental health concerns Personalized therapeutic choices based on user feedback	NR^b^
Wysa (Inkster et al, 2018) [45]	Wellbeing support app for users with symptoms of depression, aiming to build mental resilience and promote mental wellbeing	Explicit: User responses to built-in assessment questionnaire and emotions expressed in a written conversation	Individuated	Personalized conversational pathways based on a user’s interaction, messages, and context	NR
Reflection Companion (Kocielnik et al, 2018) [46]	Support reflection on personal physical activity data from fitness trackers	Explicit: Users enter their behavior change goals and demographic data Implicit: Observed physical activity of the user	Individuated	Dialogues to encourage reflection Incorporating user goals into adaptive mini-dialogues Follow-up questions based on users’ earlier responses Visualization of past physical activity	NR
Relational Agent (Sillice et al, 2018) [47]	Promote regular exercising and sun protection	Explicit: Users provide their demographic information, exercising habits, sun protection behaviors and lifestyle goals Implicit: CA tracks user progress to send reminders if needed	Individuated	Acknowledgement of difficulties and tailored strategies to overcome these Feedback on progress and encouragement for achieving goals A weekly tracking chart to help participants monitor their exercise and sun protection behaviors Email reminders to support retention	NR
Woebot (Fitzpatrick et al, 2017) [35]	Deliver cognitive-behavioral therapy for anxiety and depression to college students	Explicit: Users enter their mood and goals	Individuated	Empathic responses tailored to the reported mood Tailoring of support content depending on the reported mood Daily prompting messages to initiate a conversation Weekly charts depicting the reported mood and textual summary	NR
Social Skills Trainer (Tanaka et al, 2017) [36]	Social skills training for people with autism spectrum disorders	Implicit: CA analyzes the user's audio-visual features, facial expression (smile), and head position to determine its feedback and then performs feature selection	Individuated	Personalized score showing similarity to a role model with respect to 10 features Encouraging comments to reinforce motivation, based on features closest to the model Comments on the points that need improvement, based on features dissimilar to the model Homework challenges for participants to complete on their own time throughout the week	NR
mASMAA^c^ (Rhee et al, 2014) [37]	Facilitate asthma symptom monitoring, treatment adherence, and adolescent-parent partnership	Explicit: Users enter symptoms, activity level, and use of rescue and control medications	Individuated	Automated inquiries and reminders sent according to user-defined preferences on monitoring symptoms and managing medications and activity Processing of and responses to user-initiated messages at any time Daily report summarizing symptoms, activity, and use emailed to parents	NR
Chris (Hudlicka, 2013) [38]	Embodied CA that provides mindfulness training and coaching	Explicit: Users answer questions asked by the CA and set preferences via multiple-choice questions	Individuated	CA’s facial expressions and its responses adapting to the users’ learning needs and motivational state CA's affective reaction adapting to the users' utterances Conversational expressions communicating mental state Customized advice about meditation practice, based on the expressed concerns	Using didactic, relational, or motivational conversational styles according to the user models
DI@l-log (Harper et al, 2008; Black et al, 2005) [39,40]	Voice logbook to document home monitored data by diabetes patients	Explicit: Users provide weight, blood sugar and blood pressure values	Individuated	An alert feature generating a verbal warning if readings are too high Personalized feedback to patients on their current progress	NR
Pain Monitoring Voice Diary (Levin and Levin, 2006) [41]	Real-time collection of information from patients for health, behavioral, and lifestyle studies and monitoring	Explicit: Users answer a series of questions about their pain (location, type, intensity, etc) Implicit: CA utilizes previous sessions to provide personalized content and conversational style	Individuated Categorical (novice and experienced users)	Content (what data is collected) and style (how it is collected) of the reporting session Adaptive question-asking (additional questions for follow-ups to sessions with high levels of pain) Adaptive interruptions to better support experienced users	Adaptive conversational style (eg, shorter question formats for follow-up sessions)
Intelligent dialogue system (Giorgino et al, 2004; Azzini et al, 2003) [42,43]	Home care and data acquisition from hypertension patients	Explicit: Users answer questions about heart rate, pressure, weight, compliance, and more Implicit: CA changes its behavior depending on the progress of the current call and the clinical history of the caller	Individuated	The questions to be asked were determined by user profiles Gives advice on recommended health behavior and next visits Issues alerts and prompts	NR

^a^CA: conversational agent.

^b^NR: not reported.

^c^mASMAA: mobile phone-based asthma self-management aid.

**Table 3:** Personalization purpose, evaluation, and outcomes in the included studies.

Conversational agent (author, year)	Personalization		
	Purpose	Evaluation	Outcomes
Tess (Fulmer et al, 2018) [44]	To improve depression and anxiety symptoms To provide more engaging and convenient user experience To provide appropriate response and strategies based on the users’ reported emotion and health concerns	Questionnaires to measure depression (PHQ-9^a^) [48], anxiety (GAD-7^b^) [49], and affect (PANAS^c^) [50] Custom-built user satisfaction questionnaire Number of messages to measure user engagement	Significantly lower depression (*P*=.03) and anxiety scores (Group 1, *P*=.045; Group 2, *P*=.03) and significant differences in the positive and negative affects (*P*=.03; sm^d^) 86% (43/50) of participants satisfied with CA^e^ (sm) Comparable levels of daily engagement (bm^f^)
Wysa (Inkster et al, 2018) [45]	To develop positive self-expression and create a responsive self-reflection environment To encourage users to build emotional resilience skills	Questionnaire to measure depression (PHQ-9) Thematic analysis of the responses to the in-app feedback questions User engagement through analysis of raised objections and thematic analysis of in-app feedback	Significant reduction in depression scores in both high (*P*<.001) and low user groups (*P*=.01; sm) 67% (191/282) of users reporting on positive app experience (sm) More than 99% (6555/6611) of detected objections were correct (bm)
Reflection Companion (Kocielnik et al, 2018) [46]	To trigger deeper reflection, which would increase motivation, empowerment, and adoption of new behavior To provide engaging, novel, and diverse conversations around reflection	Questionnaires to measure health awareness [51], mindfulness (FMI^g^) [52], and reflection (RQ^h^) [53] Willingness to use the system, number, and length of responses as measures of engagement Responses to mini-dialogues Semi-structured post-study interviews	Significant increases in habitual action (*P*=.05) and understanding (*P*=.07; sm) Prolonged use of CA (additional two weeks) by half of the participants (16/33) with an avg of 98.4-character response length in this period (bm) High response rates: 96% (443/462) of initial and 90% (386/429) of follow-up questions (bm) Mini-dialogues successfully supporting discussions on awareness related to goal accomplishment, self-tracking data, and trends in behaviour (nq^i^, sm) Interviews indicating an increase in awareness, mindfulness, and motivation; understanding of alternatives and actions; and newly discovered insights (sm)
Relational Agent (Sillice et al, 2018) [47]	To increase user engagement and promote more effective behavior change To monitor exercise and sun protection behavior To provide strategies to overcome the reported barriers	Interviews to assess user experience and a 10-point Likert scale to measure satisfaction with interventions	The levels of satisfaction ranged between 7 and 10 on a scale of 1 to 10 (sm) Most participants reporting on: (1) positive interactions with the CA (32/34; 94%); (2) tailored feedback supporting regular exercising and sun protection behaviors (29/34; 85%); and (3) email reminders helping to remain on track with the program (23/34; 68%; sm)
Woebot (Fitzpatrick et al, 2017) [35]	To engage individuals with CA through managing conversation tailored to the reported mood	Questionnaires to measure depression (PHQ-9), anxiety (GAD-7), and affect (PANAS) Custom-built questionnaire to measure user satisfaction, emotional awareness, learning, and relevancy of content	Significant reduction in depression symptoms (*P*=.04; sm) Significantly high level of overall satisfaction (*P*<.001) and greater amount of emotional awareness (*P*=.02; sm)
Social Skills Trainer (Tanaka et al, 2017) [36]	To provide personalized feedback aimed at improving narrative social skills	Experienced human social skills trainer assessed the participants' narrative skills	Improvements in the overall narrative and social skills (Study 1, *P*=.03; Study 2, *P*=.003; bm)
mASMAA^j^ (Rhee et al, 2014) [37]	To make the system more appealing and elicit greater and longer interest in and use of the system	Six routine asthma-diary questions Focus group interviews to evaluate user experience with CA	Improved self-management, treatment adherence, accessibility of advice, awareness of symptoms, and sense of control (nq, sm) CA was found to be easy-to-use, convenient, and appealing (nq, sm)
Chris (Hudlicka, 2013) [38]	To deepen the relationship with the user To support pedagogical strategies necessary for effective training of mindfulness meditation To provide the coaching required to initiate and maintain regular practice To provide interactions for maintaining motivation via empathic dialogue and customized advice	Custom-built questionnaires to assess the overall experience, meditation frequency, knowledge of mindfulness, sense of self-efficacy, and stages of change within the transtheoretical model of change	Improved outcomes with CA group compared to a self-administered program: (1) more frequent and longer mindfulness training sessions (*P*=.01); (2) more rewarding, enjoyable, beneficial, and engaging experience (nq); and(3) more advanced stages of change and more confidence in ability to maintain regular meditation (nq) Neutral to mildly positive feedback on CA's ability to provide customized feedback (0.3 on a –2 to +2 Likert scale; sm)
DI@l-log (Harper et al, 2008; Black et al, 2005) [39,40]	To provide personalized feedback on the patient's health status and increase their engagement	Task completion rate and time Number of personalized alerts Qualitative interviews	92.2% (190/206) successfully completed calls, shortening calls over time, and effective alerts leading to 12 therapeutic interventions (bm) [39] 90.4% (38/42) successfully completed calls, users’ appreciation of the personalization and reports on empowerment, peace-of-mind, and sense of care (bm, sm) [40]
Pain Monitoring Voice Diary (Levin and Levin, 2006) [41]	To shorten the dialogue sessions To provide the users a feeling of continuity To have flexible and adaptive support for different types of users	Session length, completion rate, and turn duration Ratio of prompt interruptions by users	97% (171/177) of sessions completed with 98% (849/859) input accuracy (bm) Shortening dialogues over time (avg 1.2 seconds over 7 sessions; bm) More prompt-interruptions by the experienced users (73% of the prompts) compared to the novice users (59% of the prompts; bm)
Intelligent dialogue system (Giorgino et al, 2004; Azzini et al, 2003) [42,43]	To improve the quality of system dialogues To increase patient compliance with guidelines	Reliability and recognition error rate Time spent in learning to use the system	Recognition rate up to 41%-81% (bm) Dialogue time of 3.3-5.9 minutes, with 80% (74/93) of the expert users’ dialogues achieving conclusion (bm)

^a^PHQ-9: Patient Health Questionnaire 9-item scale.

^b^GAD-7: Generalized Anxiety Disorder 7-item scale.

^c^PANAS: positive and negative affect schedule 20-item scale.

^d^sm: self-reported measure.

^e^CA: conversational agent.

^f^bm: behavioral measure.

^g^FMI: Freiburg Mindfulness Inventory.

^h^RQ; Reflection Questionnaire.

^i^nq: not quantified.

^j^mASMAA: mobile phone-based asthma self-management aid.

### What is Personalized?

Personalization was primarily used for tailoring the content to be delivered. Personalized content included: (1) feedback on mood states [35], narrative skills [36], symptom summaries [37], meditation practice [38], and current progress towards the goals set [39,40,46,47]; (2) reminders [37,47], warnings, and alerts [39,40,42,43]; (3) multimedia [35,46]; and (4) questions on pain [41], physical activity [46], and health status [42,43].

Two studies personalized the user interface through changing conversational styles according to users’ motivation state, users’ level of expertise with the system, and dialogue history [38,41]. For example, one study used either didactic, relational, or motivational conversational styles based on the user profile and progress [38]. While the didactic style was used for training-related conversations, the relational style was used at the beginning of sessions to improve user engagement based on the answers received from the user. The motivational style was employed to gather progress-related information and then to provide customized responses to support users. In a simpler implementation, another study used shorter question formats for follow-up sessions [41].

The purposes of providing personalized content and conversations were to: (1) improve user engagement [35,37,38] and dialogue quality [42,43,54]; (2) provide timely feedback [39,40], adaptive user support [41], and adaptive training [36,38]; and, (3) support self-reflection [45,46].

### Evaluation of Personalization

Only two studies directly assessed users’ perceptions of personalization via custom-built questionnaires with questions on adaptive features [38] or via interview questions on tailored feedback [47]. One study employed a virtual coach to teach mindfulness and meditation [38]. The intervention group participants found the experience more rewarding, enjoyable, beneficial, and engaging than the control group participants. The coach’s ability to provide customized feedback was the most successful feature, but this was only rated neutral to mildly positive (0.3 on a –2 to +2 Likert scale). Another study evaluated a relational agent to promote exercise and sun protection [47], with a total of 85% (29/34) of the study participants finding the tailored feedback helpful for achieving their behavior change goals [47]. The remaining studies did not directly evaluate the personalized features. Rather, they focused on evaluating factors that could be associated with personalization, such as user satisfaction, user engagement, and dialogue quality, or effects of personalization, such as improved skills, self-management, and awareness of the user’s health status. One study conducted user interviews in which the users made positive remarks on personalization features [40].

## Discussion

### Principal Results

The use of CAs with unconstrained language input in health care is still limited, but there has been a notable increase in the number of studies in recent years. Almost half of the papers included in this study were published in the last two years. While most studies used quasi-experimental study designs, only two used randomized controlled trials [35,44]. Considering the recent emphasis on the role of replication in health informatics [55], the lack of technical details on conversational systems used in the studies is a major obstacle impeding replicability. In terms of personalization, our review found only 13 studies with personalization features. The studies provided various forms of personalized content, however, they were implemented without being supported by any prior evidence showing their effectiveness or any theoretical frameworks underpinning personalization [7,56,57]. Only three studies explicitly mentioned utilization of user profiles or user models to support personalized and adaptive features [42,43,46]. Similarly, only two studies directly assessed the personalization features [38,47]. The effects of the chosen personalization methods (either implicit, explicit, or a mix of the two) on user engagement and health outcomes received little attention.

While personalization of content to be delivered was common across all the studies, personalization of conversational style was implemented by only two studies [38,41]. The lack of conversational adaptation can be an impediment to improving usability and user experience, since different users may have different conversational preferences and needs that require different conversational strategies to be applied. Previous research has shown that adaptive conversational strategies can improve system performance, usability, and efficiency [58,59]. In this review, examples of conversational adaptation included using shorter questions for follow-up sessions [41] or using didactic, relational, or motivational conversational styles according to the user models [38]. Although such adaptive behaviors are useful steps towards accommodating the needs of various users, there are advanced implementations of conversational adaptation that can be applied to health care CAs, such as implicitly detecting users’ level of expertise and thus adjusting the complexity of the terms used and the dialogue path to be taken [60], or configuring the level of system initiative and confirmation strategies when a user faces difficulties in performing a task [12].

Only two studies evaluated personalization as a distinct factor [38,47]. The direct assessment of personalization, involving how users perceive the extent of personalization, is an important element in the evaluation processes. When the effects of personalization were evaluated, it was not possible to determine whether any of the outcomes were attributed to the availability of personalization features or to other factors. Therefore, new conversational agent studies with carefully controlled conditions are needed to understand the relationships between personalization features and other evaluation factors such as user satisfaction and user engagement. To guide the direct assessment of personalization, a theoretical framework such as the one developed by Fan and Poole [7] may prove useful for systematically considering various dimensions of the personalization process.

The implications of different implementations of personalization were not addressed by any studies. For example, a recent research paper drew attention to the limitations of implicit and explicit personalization [61]: while implicit personalization with its often-imperceptible user models and hidden assumptions can result in biased decision-making [62], over-reliance on system suggestions [63], and filter bubbles [64], explicit personalization may involve very formulaic and superficial choices for users who may not be well-equipped to customize the presented choices in a satisfactory manner [65]. The study employed a reflective personalization approach, allowing users to reflect on their own goals and priorities when making or modifying choices [61]. This approach demonstrated an implementation of personalization that recognizes the complexities associated with human choices, preferences, and agency when using interactive technologies. Out of all the studies in our review, one study implemented a reflective approach to personalization by using adaptive mini-dialogues to support users’ self-reflections on their goals [46]. These dialogues were successful in supporting discussions on awareness, goal accomplishment, self-tracking data, and trends in behavior.

Using CAs with unconstrained natural language input can be risky [66]. Thus, it is important for such CAs to include patient harm considerations into their study protocols. None of the included studies reported any personalization-related harms., but there was a lack of attention to the safety implications of CAs, as evident in the absence of patient safety as an evaluation dimension. In addition to patient safety, future studies need to consider the effects of different personalization methods on patient privacy. In particular, implicit methods used for gathering user information need to be clearly communicated to the users, since such methods often run automatically in the background, not being noticed by users. To this end, the model of informed consent for information systems may prove useful for considering various factors involved in collecting personal information [67].

Overall, most of the reviewed papers did not focus explicitly on personalization. Little attention was generally paid to the complexities associated with implementing personalization features and measuring their effects.

### Comparison with Prior Work

In line with our study, a recent scoping review of psychology-focused embodied conversational agents reported that only a few studies employed user models to personalize user-system interactions [68]. Another recent mapping study on health chatbots for behavior change found personalization to be one of the most appreciated technical enablers [69]. In terms of the implementation of personalization features, most of the studies in our review implemented personalization features without being informed by the advancements in other domains of personalization (eg, more automated personalization methods [12,60] or the implications of personalization on privacy, safety, and decision-making [61,62,70]).

### Limitations

Our results are based on the presence of personalization features of health care CAs in the studies that do not necessarily have an explicit focus on personalization. Therefore, the results are limited by the extent to which the included studies reported on their personalization features. In addition, our review focused on CAs using unconstrained natural language input. Therefore, the results may not be extended to agents using constrained natural language input (eg, multiple-choice of utterance options). Since the conversational systems used in the reviewed studies involved multiple components, the reported outcomes were attributable to the systems rather than only the personalization features. Our paper recommended using a theoretical framework of personalization to support a more systematic treatment of personalization features. However, it may be possible to implement personalization features effectively with no theoretical support. Moreover, other theories not specific to personalization may prove useful for personalization purposes, such as the Theory of Planned Behavior [71]. Various contextual factors such as location and time may also be integrated with user models to support more adaptive information and services [72].

### Future Research Directions

Future research can focus on incorporating a theoretical framework [7] and an evidence-based approach to implement personalization features in the domain of health care CAs. Another line of research could investigate the relationships between personalization features in conversational systems and health processes, and outcome measures such as treatment adherence or management of chronic health conditions. Future work can also focus on the use of the unique characteristics of the conversational medium for personalization purposes, such as capturing prosodic features in users’ speech to automatically detect changes in mood or speech pathologies and thus provide adaptive information and services.

### Conclusions

The use of personalization in health care CAs with unconstrained natural language interfaces has been limited and is not evidence based. While the CAs with personalization features were reported to improve user satisfaction, user engagement, and dialogue quality, little evaluation was performed to measure the extent of personalization and its role in improving health outcomes. Future research in health care CAs could evaluate the impact of personalization on health outcomes and its potential implications on privacy, safety, and decision-making.

## Appendix

Multimedia Appendix 1 The search strategy.

Multimedia Appendix 2 The list of excluded articles.

## Abbreviations

CA: Conversational Agent
FMI: Freiburg Mindfulness Inventory
GAD: Generalized Anxiety Disorder
NR: Not reported
PANAS: Positive and Negative Affect Scale
PHQ: Patient Health Questionnaire
RQ: Reflection Questionnaire
