# Supplementary material for: The Personalization of Conversational Agents in Health Care: Systematic Review
Source: J Med Internet Res. 2019 Nov 7;21(11):e15360. doi: 10.2196/15360 (PMC6873147; doi:10.2196/15360)
Supplement: Multimedia Appendix 1 [file jmir_v21i11e15360_app1.pdf]

Appendix 1: The Search Strategy

1.1. Search strategy for MEDLINE (via PubMed interface)
(https://www.ncbi.nlm.nih.gov/pubmed/)
Filters: none
Conducted in April 2017 and updated in March 2019
"Conversational agent*" OR "conversational system*" OR "dialog system*" OR "dialogue system*" OR "assistance technology" OR "assistance technologies" OR "relational agent*" OR chatbot*

1.2. Search strategy for EMBASE
URL: Macquarie University Library (via OVID interface)
Limits: none
[mp=title, abstract, heading word, table of contents, key concepts, original title, tests & measures]
Conducted in April 2017 and updated in March 2019
Conversational agent*.mp OR conversational system*.mp OR dialog system*.mp OR dialogue system*.mp OR assistance technology.mp OR assistance technologies.mp OR relational agent*.mp OR chatbot*.mp

1.3. Search strategy for PsychINFO
URL: Macquarie University Library (via OVID interface)
Limits: none
[mp=title, abstract, heading word, table of contents, key concepts, original title, tests & measures]
Conducted in April 2017 and updated in March 2019
Conversational agent*.mp OR conversational system*.mp OR dialog system*.mp OR dialogue system*.mp OR assistance technology.mp OR assistance technologies.mp OR relational agent*.mp OR chatbot*.mp

1.4. Search strategy for CINAHL
URL: Macquarie University Library (via EBSCO Publishing)
Limits: none
Conducted in April 2017 and updated in March 2019
"Conversational agent*" OR "conversational system*" OR "dialog system*" OR "dialogue system*" OR "assistance technology" OR "assistance technologies" OR "relational agent*" OR chatbot*

1.5. Search strategy for ACM Digital Library
URL: Macquarie University Library (via EBSCO Publishing)
Conducted in April 2017 and updated in March 2019
"Conversational agent*"
"conversational system*"
"dialog system*"
"dialogue system*"
"relational agent*"
chatbot*
