# Supplementary material for: The Personalization of Conversational Agents in Health Care: Systematic Review
Source: J Med Internet Res. 2019 Nov 7;21(11):e15360. doi: 10.2196/15360 (PMC6873147; doi:10.2196/15360)
Supplement: Multimedia Appendix 2 [file jmir_v21i11e15360_app2.pdf]

Appendix 2: The list of excluded articles

List of articles excluded after the full-text review, for not meeting inclusion criteria regarding the population, intervention, outcome, study design or personalization. Within the excluded studies, some major themes included counselling, aged-care, education, and behavior change.

Population:

1. Green, D. and R. Cornish. *DISPLAYTRAN a graphic oriented conversational system*. in *Proceedings of the SHARE design automation project*. 1966. ACM.
2. Griol, D., J. Carbo, and J.M. Molina, *Bringing context-aware access to the web through spoken interaction.* Applied intelligence, 2013. **38**(4): p. 620-640.
3. Griol, D., et al., *A Two-Stage Combining Classifier Model for the Development of Adaptive Dialog Systems.* International journal of neural systems, 2016. **26**(01): p. 1650002.
4. Heylen, D., et al., *On the nature of engineering social artificial companions.* Applied Artificial Intelligence, 2011. **25**(6): p. 549-574.
5. Higuchi, S., R. Rzepka, and K. Araki. *A casual conversation system using modality and word associations retrieved from the web*. in *Proceedings of the Conference on Empirical Methods in Natural Language Processing*. 2008. Association for Computational Linguistics.
6. Hochberg, J., N. Kambhatla, and S. Roukos. *A flexible framework for developing mixed-initiative dialog systems*. in *Proceedings of the 3rd SIGdial workshop on Discourse and dialogue-Volume 2*. 2002. Association for Computational Linguistics.
7. Hoque, M.E., et al. *Mach: My automated conversation coach*. in *Proceedings of the 2013 ACM international joint conference on Pervasive and ubiquitous computing*. 2013. ACM.
8. L'Abbate, M. and U. Thiel. *Helping conversational agents to find informative responses: query expansion methods for chatterbots*. in *Proceedings of the first international joint conference on Autonomous agents and multiagent systems: part 2*. 2002. ACM.
9. Li, J., et al. *Confiding in and Listening to Virtual Agents: The Effect of Personality*. in *Proceedings of the 22nd International Conference on Intelligent User Interfaces*. 2017. ACM.
10. Litman, D., et al. *NJFun: a reinforcement learning spoken dialogue system*. in *Proceedings of the 2000 ANLP/NAACL Workshop on Conversational systems-Volume 3*. 2000. Association for Computational Linguistics.
11. Luger, E. and A. Sellen. *Like having a really bad PA: the gulf between user expectation and experience of conversational agents*. in *Proceedings of the 2016 CHI Conference on Human Factors in Computing Systems*. 2016. ACM.
12. Maier, E., N. Reithinger, and J. Alexandersson. *Clarification dialogues as measure to increase robustness in a spoken dialogue system*. in *Interactive Spoken Dialog Systems on Bringing Speech and NLP Together in Real Applications*. 1997. Association for Computational Linguistics.
13. Miner, A., et al. *Conversational Agents and Mental Health: Theory-Informed Assessment of Language and Affect*. in *Proceedings of the Fourth International Conference on Human Agent Interaction*. 2016. ACM.
14. Nederhof, M.-J., et al. *Grammatical analysis in the OVIS spoken-dialogue system*. in *Interactive Spoken Dialog Systems on Bringing Speech and NLP Together in Real Applications*. 1997. Association for Computational Linguistics.

15. O'Neill, I.M. and M.F. McTear. *An object-oriented model for the design of cross-domain dialogue systems*. in *Interactive Spoken Dialog Systems on Bringing Speech and NLP Together in Real Applications*. 1997. Association for Computational Linguistics.
16. Filichia, L., Halan, S., Blackwelder, E., Rossen, B., Lok, B., Korndorffer, J., & Cendan, J. *Description of web-enhanced virtual character simulation system to standardize patient hand-offs*. Journal of Surgical Research. 2011. 176-181.

Intervention:

17. Auriacombe M, Moriceau S, Serre F, et al. *Development and validation of a virtual agent to screen tobacco and alcohol use disorders*. Drug and alcohol dependence 2018;193:1-6.
18. Baker, S., D. Richards, and P. Caldwell. *Putting a New Intelligent Virtual Face on a Medical Treatment Advice System to Improve Adherence*. in *Proceedings of the 2014 Conference on Interactive Entertainment*. 2014. ACM.
19. Barker, D.J., et al., *Evaluating a spoken dialogue system for recording clinical observations during an endoscopic examination.* Med Inform Internet Med, 2003. **28**(2): p. 85-97.
20. Berry, D.C., L.T. Butler, and F. De Rosis, *Evaluating a realistic agent in an advice-giving task.* International Journal of Human-Computer Studies, 2005. **63**(3): p. 304-327.
21. Bickmore, T. and T. Giorgino, *Health dialog systems for patients and consumers.* Journal of biomedical informatics, 2006. **39**(5): p. 556-571.
22. Bickmore, T. and A. Gruber, *Relational agents in clinical psychiatry.* Harvard review of psychiatry, 2010. **18**(2): p. 119-130.
23. Bickmore, T., A. Gruber, and R. Picard, *Establishing the computer–patient working alliance in automated health behavior change interventions.* Patient education and counseling, 2005. **59**(1): p. 21-30.
24. Bickmore, T., D. Schulman, and L. Yin, *Maintaining engagement in long-term interventions with relational agents.* Applied Artificial Intelligence, 2010. **24**(6): p. 648-666.
25. Bickmore, T.W., *Relational agents: Effecting change through human-computer relationships*, 2003, Massachusetts Institute of Technology.
26. Bickmore, T.W., et al., *'It's just like you talk to a friend'relational agents for older adults.* Interacting with Computers, 2005. **17**(6): p. 711-735.
27. Bickmore, T.W., et al., *Response to a relational agent by hospital patients with depressive symptoms.* Interacting with computers, 2010. **22**(4): p. 289-298.
28. Bickmore, T.W., et al., *Usability of conversational agents by patients with inadequate health literacy: evidence from two clinical trials.* Journal of health communication, 2010. **15**(S2): p. 197-210.
29. Bickmore, T.W., L.M. Pfeifer, and B.W. Jack. *Taking the time to care: empowering low health literacy hospital patients with virtual nurse agents*. in *Proceedings of the SIGCHI conference on human factors in computing systems*. 2009. ACM.
30. Bickmore, T.W., L.M. Pfeifer, and M.K. Paasche-Orlow, *Using computer agents to explain medical documents to patients with low health literacy.* Patient education and counseling, 2009. **75**(3): p. 315-320.
31. Bickmore, T.W., et al., *Maintaining reality: relational agents for antipsychotic medication adherence.* Interacting with Computers, 2010. **22**(4): p. 276-288.

32. Bickmore, T.W., D. Schulman, and C. Sidner, *Automated interventions for multiple health behaviors using conversational agents.* Patient education and counseling, 2013. **92**(2): p. 142-148.
33. Bickmore, T.W., D. Schulman, and C.L. Sidner, *A reusable framework for health counseling dialogue systems based on a behavioral medicine ontology.* Journal of biomedical informatics, 2011. **44**(2): p. 183-197.
34. Bickmore, T.W., et al., *A randomized controlled trial of an automated exercise coach for older adults.* Journal of the American Geriatrics Society, 2013. **61**(10): p. 1676-1683.
35. Bickmore, T.W., et al., *Improving access to online health information with conversational agents: a randomized controlled experiment.* Journal of medical Internet research, 2016. **18**(1).
36. Black, L., et al. *DI@ L-log By Design: An Integrated Tele-Care Communication Infrastructure*. in *Engineering in Medicine and Biology Society, 2004. IEMBS'04. 26th Annual International Conference of the IEEE*. 2004. IEEE.
37. Cafaro, A., H.H. Vilhjálmsson, and T. Bickmore, *First Impressions in Human--Agent Virtual Encounters.* ACM Transactions on Computer-Human Interaction (TOCHI), 2016. **23**(4): p. 24.
38. Callejas, Z., D. Griol, and R. López-Cózar, *A framework for the assessment of synthetic personalities according to user perception.* International Journal of Human-Computer Studies, 2014. **72**(7): p. 567-583.
39. Callejas, Z. and R. Lopez-Cozar, *Influence of contextual information in emotion annotation for spoken dialogue systems.* Speech Communication, 2008. **50**(5): p. 416-433.
40. Casas J, Mugellini E, Khaled OA. *Food Diary Coaching Chatbot. Proceedings of the 2018 ACM International Joint Conference and 2018 International Symposium on Pervasive and Ubiquitous Computing and Wearable Computers*. Singapore, Singapore: ACM, 2018:1676-80.
41. Chinaei H, Currie LC, Danks A, Lin H, Mehta T, Rudzicz F. *Identifying and avoiding confusion in dialogue with people with alzheimer's disease.* Comput. Linguist. 2017;43(2):377-406.
42. Creed, C., R. Beale, and B. Cowan, *The Impact of an Embodied Agent's Emotional Expressions Over Multiple Interactions.* Interacting with Computers, 2014. **27**(2): p. 172-188.
43. Denecke K, Hochreutener SL, P A, #246, pel, May R. *Talking to Ana: A Mobile Self-Anamnesis Application with Conversational User Interface. Proceedings of the 2018 International Conference on Digital Health.* Lyon, France: ACM, 2018:85-89.
44. Fadhil A, Schiavo G, Wang Y, Yilma BA. *The Effect of Emojis when interacting with Conversational Interface Assisted Health Coaching System. Proceedings of the 12th EAI International Conference on Pervasive Computing Technologies for Healthcare*. New York, NY, USA: ACM, 2018:378-83.
45. Ghosh S, Bhatia S, Bhatia A. Quro: Facilitating User Symptom Check Using a Personalised Chatbot-Oriented Dialogue System. Studies in health technology and informatics 2018;252:51-56.
46. Granström, B., *The use of speech synthesis in exploring different speaking styles.* Speech Communication, 1992. **11**(4-5): p. 347-355.
47. Green, N. and J.F. Lehman, *An integrated discourse recipe-based model for task-oriented dialogue.* Discourse Processes, 2002. **33**(2): p. 133-158.

48. Griol, D. and Z. Callejas, *Mobile conversational agents for context-aware care applications.* Cognitive Computation, 2016. **8**(2): p. 336-356.
49. Grundy J, Mouzakis K, Vasa R, et al. *Supporting Diverse Challenges of Ageing with Digital Enhanced Living Solutions.* Studies in health technology and informatics 2018;246:75-90.
50. Harrefors, C., K. Axelsson, and S. Sävenstedt, *Using assistive technology services at differing levels of care: healthy older couples' perceptions.* Journal of advanced nursing, 2010. **66**(7): p. 1523-1532.
51. Hasegawa, S., et al. *Multilingual medical dialog system developed as smartphone/tablet application*. in *Engineering in Medicine and Biology Society (EMBC), 2013 35th Annual International Conference of the IEEE*. 2013. IEEE.
52. Ho A, Hancock J, Miner AS. *Psychological, Relational, and Emotional Effects of Self-Disclosure After Conversations with a Chatbot*. The Journal of communication 2018;68(4):712-33.
53. Holter, M.T., A. Johansen, and H. Brendryen, *How a fully automated eHealth program simulates three therapeutic processes: a case study.* Journal of medical Internet research, 2016. **18**(6).
54. Hsu P, Zhao J, Liao K, Liu T, Wang C. *AllergyBot: A Chatbot Technology Intervention for Young Adults with Food Allergies Dining Out. Proceedings of the 2017 CHI Conference Extended Abstracts on Human Factors in Computing Systems*. Denver, Colorado, USA: ACM, 2017:74-79.
55. Hubal, R.C., et al., *How do varied populations interact with embodied conversational agents? Findings from inner-city adolescents and prisoners.* Computers in human behavior, 2008. **24**(3): p. 1104-1138.
56. Jack, B., et al., *Reducing preconception risks among African American women with conversational agent technology.* The Journal of the American Board of Family Medicine, 2015. **28**(4): p. 441-451.
57. Kim, J.H., et al., *A web-based rapid prototyping and clinical conversational system that complements electronic patient record system.* Medinfo, 2001. **10**: p. 628-632.
58. Korpusik M, Glass J, Korpusik M, Glass J. *Spoken Language Understanding for a Nutrition Dialogue System*. IEEE/ACM Trans. Audio, Speech and Lang. Proc. 2017;25(7):1450-61.
59. López, B., et al. *Design and validation of ECA gestures to improve dialogue system robustness*. in *Proceedings of the Workshop on Embodied Language Processing*. 2007. Association for Computational Linguistics.
60. Lacson, R., *The medical appointment scheduler.* Studies in health technology and informatics, 2004. **107**(Pt 2): p. 969.
61. Lin, C.-J., et al., *Ellipsis and Coreference Resolution in a Computerized Virtual Patient Dialogue System.* Journal of medical systems, 2016. **40**(9): p. 206.
62. Lisetti, C., et al., *I can help you change! an empathic virtual agent delivers behavior change health interventions.* ACM Transactions on Management Information Systems (TMIS), 2013. **4**(4): p. 19.
63. Mairittha T, Okita T, Inoue S. *Pre-Consulting Dialogue Systems for Telemedicine: Yes/No Intent Classification. Proceedings of the 2018 ACM International Joint Conference and 2018 International Symposium on Pervasive and Ubiquitous Computing* and Wearable Computers. Singapore, Singapore: ACM, 2018:742-45.
64. Martin J-C, Lescanff C, Rosset S, Walker M, Whittaker S. How to Personalize Conversational Coaches for Stress Management? Proceedings of the 2018 ACM

International Joint Conference and 2018 International Symposium on Pervasive and Ubiquitous Computing and Wearable Computers. Singapore, Singapore: ACM, 2018:718-21.

65. McRoy, S., et al. *An open architecture for messaging-based consumer-health question-answering*. in *Proceedings of the 2nd ACM SIGHIT International Health Informatics Symposium*. 2012. ACM.
66. Mehrotra, S., et al. *Embodied Conversational Interfaces for the Elderly User*. in *Proceedings of the 8th Indian Conference on Human Computer Interaction*. 2016. ACM.
67. Morris RR, Kouddous K, Kshirsagar R, Schueller SM. *Towards an Artificially Empathic Conversational Agent for Mental Health Applications: System Design and User Perceptions*. Journal of medical Internet research 2018;20(6):1-1.
68. Na, #273, Terzimehi a, et al. *Lunchocracy: Improving Eating Dynamics in the Workplace Using a Bot-Based Anonymous Voting System. Extended Abstracts of the 2018 CHI Conference on Human Factors in Computing Systems*. Montreal QC, Canada: ACM, 2018:1-6.
69. Novielli, N., et al., *Analysing user's reactions in advice*-giving *dialogues with a socially intelligent ECA.* Cognitive processing, 2012. **13**(2): p. 487-497.
70. Pickard, M.D., *Persuasive embodied agents: Using embodied agents to change people's behavior, beliefs, and assessments*2012: The University of Arizona.
71. Pirolli P, Youngblood G, Du H, Konrad A, Nelson L, Springer A. *Scaffolding the mastery of healthy behaviors with fittle+ systems: Evidence-based interventions and theory*. Human-Computer Interaction 2018
72. Puskar, K., et al., *Relational agents as an adjunct in schizophrenia treatment.* Journal of psychosocial nursing and mental health services, 2011. **49**(8): p. 22-29.
73. Richards D, Caldwell P. *Improving Health Outcomes Sooner Rather Than Later via an Interactive Website and Virtual Specialist*. IEEE journal of biomedical and health informatics 2018;22(5):1699-706.
74. Ring, L., T. Bickmore, and P. Pedrelli, *Real-Time Tailoring of Depression Counseling by Conversational Agent.* Iproceedings, 2016. **2**(1): p. e27.
75. de Rosis, F., et al., *User modeling and adaptation in health promotion dialogs with an animated character.* Journal of biomedical informatics, 2006. **39**(5): p. 514-531.
76. Sakpal, R. *Virtual patients to teach cultural competency*. in *Proceedings of the 14th ACM international conference on Multimodal interaction*. 2012. ACM.
77. Shamekhi, A., et al. *A Virtual Self-care Coach for Individuals with Spinal Cord Injury*. in *Proceedings of the 18th International ACM SIGACCESS Conference on Computers and Accessibility*. 2016. ACM.
78. Sidner CL, Bickmore T, Nooraie B, et al. *Creating New Technologies for Companionable Agents to Support Isolated Older Adults.* ACM Trans. Interact. Intell. Syst. 2018;8(3):1-27 doi: 10.1145/3213050
79. Simon, S.R., et al. *A Pilot Study Of A Computer-Based Relational Agent To Screen For Substance-Use Problems In Primary Care*. In *Journal Of General Internal Medicine*. 2013. Springer 233 Spring St, New York, Ny 10013 Usa.
80. Sinoo C, van der Pal S, Blanson Henkemans OA, et al. *Friendship with a robot: Children's perception of similarity between a robot's physical and virtual embodiment that supports diabetes self-management.* Patient education and counseling 2018
81. Soller, R.W., P. Chan, and A. Higa, *Performance of a new speech translation device in translating verbal recommendations of medication action plans for patients with diabetes.* Journal of diabetes science and technology, 2012. **6**(4): p. 927-937.

82. Suganuma S, Sakamoto D, Shimoyama H. *An Embodied Conversational Agent for Unguided Internet-Based Cognitive Behavior Therapy in Preventative Mental Health: Feasibility and Acceptability Pilot Trial*. JMIR mental health 2018;5(3):e10454 doi: 10.2196/10454.
83. Sun O, Chen J, Magrabi F. *Using Voice-Activated Conversational Interfaces for Reporting Patient Safety Incidents: A Technical Feasibility and Pilot Usability Study*. Studies in health technology and informatics 2018;252:139-44.
84. Tiwari, P., J. Warren, and K. Day. *Empowering older patients to engage in self care: designing an interactive robotic device*. in *AMIA Annual Symposium Proceedings*. 2011. American Medical Informatics Association.
85. Waller, A., et al., *Evaluating the use of TalksBac, a predictive communication device for nonfluent adults with aphasia.* International Journal of Language & Communication Disorders, 1998. **33**(1): p. 45-70.
86. Wang, C., et al., *Acceptability and feasibility of a virtual counselor (VICKY) to collect family health histories.* Genetics in medicine: official journal of the American College of Medical Genetics, 2015. **17**(10): p. 822.
87. Wang V, Wexler S, Drury L, Wang B. *A Protocol-Driven, Digital Conversational Agent at the Hospital Bedside to Support Nurse Teams and to Mitigate Delirium and Falls Risk*. Journal of medical Internet research 2018;20(9):3-3.
88. Wargnier P, Benveniste S, Jouvelot P, Rigaud AS. Usability assessment of interaction management support in LOUISE, an ECA-based user interface for elders with cognitive impairment. Technology and Disability 2018;30(3):105-26
89. Wells, K.J., et al., *Acceptability of a virtual patient educator for hispanic women.* Hispanic Health Care International, 2015. **13**(4): p. 179-185.
90. Wong, W., J. Thangarajah, and L. Padgham. *Health conversational system based on contextual matching of community-driven question-answer pairs*. in *Proceedings of the 20th ACM international conference on Information and knowledge management*. 2011. ACM.
91. Wright-Berryman, J.L., et al., *Consumer and provider responses to a computerized version of the Illness Management and Recovery Program.* Psychiatric rehabilitation journal, 2013. **36**(4): p. 231.
92. Xu, J., et al., *Design challenges and guidelines for persuasive technologies that facilitate healthy lifestyles.* Computer technology and application, 2012. **3**(2): p. 140.
93. Zhang Z, Bickmore T. *Medical Shared Decision Making with a Virtual Agent. Proceedings of the 18th International Conference on Intelligent Virtual Agents*. Sydney, NSW, Australia: ACM, 2018:113-18.
94. Zhang, Z., T.W. Bickmore, and M.K. Paasche-Orlow, *Perceived organizational affiliation and its effects on patient trust: Role modeling with embodied conversational agents.* Patient Education and Counseling, 2017.
95. Zhou, S., et al. *Afraid to ask: proactive assistance with healthcare documents using eye tracking*. in *Proceedings of the extended abstracts of the 32nd annual ACM conference on Human factors in computing systems*. 2014. ACM.
96. van der Zwaan, J.M. and V. Dignum. *Robin, an empathic virtual buddy for social support*. in *Proceedings of the 2013 international conference on Autonomous agents and multi-agent systems*. 2013. International Foundation for Autonomous Agents and Multiagent Systems.

Outcome:

97. Allen, J., et al., *Chester: towards a personal medication advisor.* Journal of biomedical informatics, 2006. **39**(5): p. 500-513.
98. Azzini, I., et al., *Automated spoken dialog system for home care and data acquisition from chronic patients.* Studies in health technology and informatics, 2003. **95**: p. 146-151.
99. Bickmore, T. *Towards the design of multimodal interfaces for handheld conversational characters*. in *CHI'02 extended abstracts on Human factors in computing systems*. 2002. ACM.
100. Bickmore, T. and J. Cassell. *Relational agents: a model and implementation of building user trust*. in *Proceedings of the SIGCHI conference on Human factors in computing systems*. 2001. ACM.
101. Bohle S. "Plutchik": *Artificial intelligence chatbot for searching NCBI databases*. Journal of the Medical Library Association 2018;106(4):501-03 doi: 10.5195/jmla.2018.500.
102. Clavel l, Whittaker S, et al. WEnner: A Theoretically Motivated Approach for Tailored Coaching about Physical Activity. Proceedings of the International Symposium on Pervasive and Ubiquitous Computing and Wearable Computers. Singapore, Singapore: ACM, 2018:1669-75.
103. Fadhil A, Gabrielli S. *Addressing challenges in promoting healthy lifestyles: the al-chatbot approach. Proceedings of the 11th EAI International Conference on Pervasive Computing Technologies for Healthcare*. Barcelona, Spain: ACM, 2017:261-65.
104. Gabrielli S, Marie K, Corte CD. *SLOWBot (chatbot) Lifestyle Assistant. Proceedings of the 12th EAI International Conference on Pervasive Computing Technologies for Healthcare*. New York, NY, USA: ACM, 2018:367-70.
105. Gumede, T. and M. Plauché. *Initial fieldwork for LWAZI: a telephone-based spoken dialog system for rural South Africa*. in *Proceedings of the First Workshop on Language Technologies for African Languages*. 2009. Association for Computational Linguistics.
106. Haimowitz, I.J., *Modeling all dialogue system participants to generate empathetic responses.* Computer Methods and Programs in Biomedicine, 1991. **35**(4): p. 321-330.
107. Kamali ME, Angelini L, Caon M, Andreoni G, Khaled OA, Mugellini E. *Towards the NESTORE e-Coach: a Tangible and Embodied Conversational Agent for Older Adults. Proceedings of the 2018 ACM International Joint Conference and 2018 International Symposium on Pervasive and Ubiquitous Computing and Wearable Computers*. Singapore, Singapore: ACM, 2018:1656-63.
108. Kim J, Kim Y, Kim B, Yun S, Kim M, Lee J. *Can a Machine Tend to Teenagers' Emotional Needs?: A Study with Conversational Agents. Extended Abstracts of the 2018 CHI Conference on Human Factors in Computing Systems*. Montreal QC, Canada: ACM, 2018:1-6.
109. Knuepffer, C. *Chat-Bots for People with Parkinson's Disease: Science Fiction or Reality?* in *Driving Reform: Digital Health is Everyone's Business: Selected Papers from the 23rd Australian National Health Informatics Conference (HIC 2015)*. 2015. IOS Press.
110. Loisel, A., N. Chaignaud, and J.-P. Kotowicz. *Designing a Human-Computer Dialog System for Medical Information Search*. in *Proceedings of the 2007 IEEE/WIC/ACM International Conferences on Web Intelligence and Intelligent Agent Technology-Workshops*. 2007. IEEE Computer Society.

111. Motalebi N, Abdullah S. *Conversational Agents to Provide Couple Therapy for Patients with PTSD. Proceedings of the 12th EAI International Conference on Pervasive Computing Technologies for Healthcare.* New York, NY, USA: ACM, 2018:347-51.
112. Papangelis, A., F. Makedon, and R. Gatchel, *Assessing and Monitoring Post-Traumatic Stress Disorder Through Natural Interaction With an Adaptive Dialogue System.* Journal of Applied Biobehavioral Research, 2014. **19**(3): p. 192-215.
113. Pasikowska, A., A. Zaraki, and N. Lazzeri. *A dialogue with a virtual imaginary interlocutor as a form of a psychological support for well-being*. in *Proceedings of the International Conference on Multimedia, Interaction, Design and Innovation*. 2013. ACM.
114. Pérez-Marín, D. and I. Pascual-Nieto, *An exploratory study on how children interact with pedagogic conversational agents.* Behaviour & Information Technology, 2013. **32**(9): p. 955-964.
115. Pilato, G., R. Pirrone, and R. Rizzo, *A kst-based system for student tutoring.* Applied Artificial Intelligence, 2008. **22**(4): p. 283-308.
116. Skowron, M., et al., *Applying a text-based affective dialogue system in psychological research: case studies on the effects of system behaviour, interaction context and social exclusion.* Cognitive Computation, 2014. **6**(4): p. 872-891.
117. Terzis, V., C.N. Moridis, and A.A. Economides, *The effect of emotional feedback on behavioral intention to use computer based assessment.* Computers & Education, 2012. **59**(2): p. 710-721.
118. Vaira L, Bochicchio MA, Conte M, Casaluci FM, Melpignano A. *MamaBot: A System based on ML and NLP for supporting Women and Families during Pregnancy*. *Proceedings of the 22nd International Database Engineering & Applications Symposium*. Villa San Giovanni, Italy: ACM, 2018:273-77.

Study design:

119. Johnson, L. *Interaction with embodied conversational agents*. in *Proceedings of the 10th international conference on Intelligent user interfaces*. 2005. ACM.
120. Kavakli, M., et al., *Tools for eMental-Health: A Coping Processor for Adaptive and Interactive Mobile Systems for Stress Management*, in *Integrating Technology in Positive Psychology Practice*2016, IGI Global. p. 127-160.
121. Kramer JN, Kunzler F, Mishra V, et al. *Investigating Intervention Components and Exploring States of Receptivity for a Smartphone App to Promote Physical Activity: Protocol of a Microrandomized Trial*. JMIR research protocols 2019;8(1):e11540 doi: 10.2196/11540
122. Lisetti, C., *Features for culturally appropriate avatars for behavior-change promotion in at-risk populations.* Stud Health Technol Inform, 2009. **144**: p. 22-6.
123. Matthias, A., *Robot lies in health care: when is deception morally permissible?* Kennedy Institute of Ethics Journal, 2015. **25**(2): p. 169-162.
124. Migneault, J.P., et al., *How to write health dialog for a talking computer.* Journal of biomedical informatics, 2006. **39**(5): p. 468-481.
125. Powers, D.M., et al. *MANA for the Ageing*. in *Proceedings of the 2010 Workshop on Companionable Dialogue Systems*. 2010. Association for Computational Linguistics.
126. Riccardi, G. *Towards healthcare personal agents*. in *Proceedings of the 2014 Workshop on Roadmapping the Future of Multimodal Interaction Research including Business Opportunities and Challenges*. 2014. ACM.

127. Rojas-Barahona, L.M. and T. Giorgino. *AdaRTE: Adaptable Dialogue Architecture and Runtime Engine-a New Architecture for Health-care Dialogue Systems*. in *Medinfo 2007: Proceedings of the 12th World Congress on Health (Medical) Informatics; Building Sustainable Health Systems*. 2007. IOS Press.
128. Rojas-Barahona, L.M. and T. Giorgino, *Adaptable dialog architecture and runtime engine (AdaRTE): a framework for rapid prototyping of health dialog systems.* international journal of medical informatics, 2009. **78**: p. S56-S68.
129. Romeo T. *Automating the patient financial assistance process to improve patient satisfaction at Trinity Health.* Health Management Technology 2018;39(2):26-26
130. Schlangen, D. *The future of spoken dialogue systems is in their past: long-term adaptive, conversational assistants*. in *NAACL-HLT Workshop on Future directions and needs in the Spoken Dialog Community: Tools and Data*. 2012. Association for Computational Linguistics.
131. Sidner, C.L., et al. *A Robotic Companion for Social Support of Isolated Older Adults*. in *HRI (Extended Abstracts)*. 2015.
132. Tartaro, A. *Authorable virtual peers for children with autism*. in *CHI'07 extended abstracts on Human factors in computing systems*. 2007. ACM.
133. Thompson, D., et al., *Use of relational agents to improve family communication in type 1 diabetes: methods.* JMIR research protocols, 2016. **5**(3).
134. Torjesen I. *Sixty seconds on . . . GP chatbot.* BMJ (Clinical research ed.) 2018;362:k2897
135. Vogeley, K. and G. Bente, *"Artificial humans": Psychology and neuroscience perspectives on embodiment and nonverbal communication.* Neural Networks, 2010. **23**(8): p. 1077-1090.
136. Weaver, K., A. Komlodi, and B. Duffy. *Using an intelligent interviewer to perform cognitive assessments*. in *CHI'13 Extended Abstracts on Human Factors in Computing Systems*. 2013. ACM.
137. Wiemer-Hastings, K., et al., *Automatic classification of dysfunctional thoughts: a feasibility test.* Behavior Research Methods, 2004. **36**(2): p. 203-212.
138. Wilson C. *Is a chatbot doctor as good as a human?* New Scientist 2018;239(3185):25-25
139. Wolters, M., et al., *Being old doesn't mean acting old: How older users interact with spoken dialog systems.* ACM Transactions on Accessible Computing (TACCESS), 2009. **2**(1): p. 2.
140. Young, V., E. Rochon, and A. Mihailidis, *Exploratory analysis of real personal emergency response call conversations: considerations for personal emergency response spoken dialogue systems.* Journal of neuroengineering and rehabilitation, 2016. **13**(1): p. 97.

Personalization:

141. Beveridge M, Fox J. *Automatic generation of spoken dialogue from medical plans and ontologies*. J Biomed Inform 2006;39(5):482-99.
142. Bickmore TW, Trinh H, Olafsson S, et al. *Patient and Consumer Safety Risks When Using Conversational Assistants for Medical Information: An Observational Study of Siri, Alexa, and Google Assistant*. J Med Internet Res 2018;20(9):e11510.
143. Crutzen R, Peters GJ, Portugal SD, Fisser EM, Grolleman JJ. *An artificially intelligent chat agent that answers adolescents' questions related to sex, drugs, and alcohol: an exploratory study*. J Adolesc Health 2011;48(5):514-9.
144. Griol D, Carbo J, Lopez JM. *An Automatic Dialog Simulation Technique to Develop and Evaluate Interactive Conversational Agents*. J Appl. Artif. Intell 2013;27(9):759-80.
145. Ireland D, Atay C, Liddle J, et al. Hello Harlie: *Enabling Speech Monitoring Through Chat-Bot Conversations*. Stud Health Technol Inform 2016;227:55-60
146. Lucas GM, Rizzo A, Gratch J, et al. *Reporting mental health symptoms: breaking down barriers to care with virtual human interviewers*. Frontiers in Robotics and AI. 2017;4:51
147. Luerssen MH, Hawke T. *Virtual Agents as a Service: Applications in Healthcare*. *Proceedings of the 18th International Conference on Intelligent Virtual Agents*. Sydney, NSW, Australia: ACM, 2018:107-12.
148. Liu B, Sundar SS. *Should Machines Express Sympathy and Empathy? Experiments with a Health Advice Chatbot*. Cyberpsychology, Behavior, and Social Networking 2018;21(10):625-36.
149. Miner AS, Milstein A, Schueller S, Hegde R, Mangurian C, Linos E. *Smartphone-Based Conversational Agents and Responses to Questions About Mental Health, Interpersonal Violence, and Physical Health*. JAMA Intern Med 2016;176(5):619-25.
150. Philip P, Micoulaud-Franchi JA, Sagaspe P, et al. *Virtual human as a new diagnostic tool, a proof of concept study in the field of major depressive disorders*. Sci Rep 2017;7:42656.
151. Philip P, Bioulac S, Sauteraud A, Chaufton C, Olive JJPt, environments v. *Could a virtual human be used to explore excessive daytime sleepiness in patient*s? Presence: teleoperators and virtual environments. 2014;23(4):369-76
